# Introducing Videoconferencing on Tablet Computers in Nurse–Patient Communication: Technical and Training Challenges

**DOI:** 10.1155/2018/8943960

**Published:** 2018-10-18

**Authors:** Lisbeth O. Rygg, Hildfrid V. Brataas, Bente Nordtug

**Affiliations:** Faculty of Nursing and Health Sciences, Nord University, Bodø, Norway

## Abstract

**Background:**

This article examines personnel and patient experiences of videoconferencing (VC) trials on tablet computers between oncology certified nurses (OCNs) and patients with cancer who live at home. The study points to organizational pitfalls during the introduction process. In many different arenas, the use of VC has increased recently owing to improved Internet access and capacity. This creates new opportunities for contact between patients living at home and their nurses. Video conferencing presupposes knowledge about Internet access, training, and usability of technological equipment. The aim of this pilot study was to illuminate patients' and nurses' experiences of the technical functionality, usability, and training of tablet use in VC in primary cancer care. The results point to the drawbacks concerning the introduction of VC.

**Method:**

A pilot study with an explorative design was used to describe patients' and OCNs' experiences of technical functionality and usability of VC on tablet computers. After a three-month trial, data were gathered, focusing on both patients' and nurses' perspectives. Individual interviews with four female OCNs, aged 32–65 (mean 46), and six patients with cancer, two men and four women aged 49–78 (mean 69), were content-analyzed.

**Results:**

The analysis revealed two main categories:* network connectivity and tablet usability* and* training and educational pitfalls*.

**Conclusion:**

When planning VC implementation, the organizational leadership should consider network access and stability, as well as individualized VC training on tablets. Ensuring patient safety should also be a priority. Further research should provide knowledge of technological and educational pitfalls, and possible implications of VC on the care quality of nursing.

## 1. Introduction

The article examines personnel and patient experiences of videoconferencing (VC) trials on tablet computers between oncology certified nurses (OCNs) and patients with cancer who live at home, determining organizational pitfalls during the introduction process.

Cancer is a major health problem worldwide. In 2015, the prevalence and incidence rates in Norway were 262.900 and 32.800, respectively [1]. Many patients with cancer have high symptom burdens during and after therapy [2]. In addition, access to healthcare in rural environments can be challenging [3, 4]. To overcome such follow-up challenges for care, VC may be a solution.

Quality work, i.e., concerns and work on ensuring quality health services and quality improvement, is not new, but has evolved over the last 50 years [5, 6]. To ensure quality services, it is necessary to employ several quality approaches and measures at a variety of levels [5, 7]. Some examples are organizational, technical, and professional approaches, which promote a culture that focuses on patients' perspectives and the best ways to meet their nursing needs and the needs of their families [5]. Therefore, both nurses' and patients' perspectives should be considered when introducing new technology. In addition, patients' safety requirements should also be emphasized [8]. When implementing tele-oncology, the aim should be to deliver high-quality medical care to rural areas [9].

The use of information and communication technology (ICT) has a long history [10]. Although ICT like VC are more commonly used in surgical treatment, there are few studies on the use of VC as a tool in primary cancer care [11–13]. Accessibility to healthcare can be promoted by VC [12–14]. Knowledge of benefits and pitfalls is needed when introducing VC in primary cancer care.

Implementing the use of VC in cancer care may benefit both patients and nurses; however, there may be a need for support and training in the use of the technical equipment. Introducing VC on tablets challenges the traditional nursing organization, as well as patient safety. Sheridan (2013) points to structures, processes, and people as framework factors in the work to prevent safety risks in cancer care [15].

The aim of this pilot study was to illuminate patients' and nurses' experiences of the technical functionality, usability, and training of tablet use in VC in primary cancer care in order to determine pitfalls concerning the introduction of VC.

## 2. Methods

A pilot study with an explorative design was used to describe patients' and OCNs' experiences of the technical functionality and usability of VC on tablet computers. The pilot study [16] is a small scale study used as part of planning a major study on the use of VC on tablets in the care for patients with cancer living at home. Individual interviews were conducted from autumn 2016 to spring 2017 and analyzed by traditional content analysis methods.

### 2.1. Sample and Sampling

This pilot study was performed in three rural municipalities in Norway. The three municipalities have about 7500 inhabitants and cover an area of 3842.1 km^2^. The sample was informative [17]. Participants were recruited among OCNs and patients with cancer. Inclusion criteria of patients comprised being aged ≥ 18 years, living at home, physically and mentally able to communicate using VC on a tablet, being diagnosed with cancer, and currently receiving cancer home care. OCNs working in the three municipalities were included: the heads of the healthcare administration in each municipality selected experienced OCNs. There were four OCNs that were included, and they were asked to then select the patients. During the project period, eight patients who met the inclusion criteria were asked to voluntarily participate. Two of them did not want to participate because they felt too sick, so a total of four women and two men were included (aged 49–78 years). One was single, and five lived with their spouses. One was employed, three were retired, and two were receiving a disability pension. The mean time since diagnosis was 27 months (range = 2–87 months). The cancer diagnoses varied, and prognoses also varied from possibly being cured to a life-long or recurrent life-threatening disease.

### 2.2. Ethics

Throughout the research process, research ethics were considered in line with the Helsinki Declaration [18]. All included patients and nurses received oral and written information from the researchers about confidentiality and anonymity and provided written consent before the interviews took place. Results were presented anonymously. The Regional Committee for Medical and Health Research Ethics in Southern Norway (ref. no. 2016/968) and the Data Inspectorate (ref. no. 49571) assessed and approved this study.

### 2.3. Intervention

A VC with sound and picture between OCNs and patients using tablets was utilized during a three-month trial period. Patients and their OCN each received a tablet that had the program “Skype” installed. As the regular Skype version was not accepted by the firewall IT-security in the municipalities' healthcare systems, the program “Skype for Business” was installed on the tablets [19]. Using this program, the patient can see on the tablet if the nurse is present [20, 21]. Skype for Business does not protect health information using firewalls [22]; patients and nurses were aware of this limitation. Both the OCNs and the patients initiated contact using VC.

An IT employee affiliated with this project provided the OCNs with oral instructions about the technical use of tablet computers and Skype. Each OCN then gave patients instructions. The IT technician also assisted to ensure the VC functioned optimally. The OCNs were free to organize their work using the VC, for example, using VC at specific times or when they felt they had the opportunity. Patients were informed that the OCNs were available during work hours from 7:00 am to 3:00 pm Monday through Friday.

### 2.4. Data Collection

Data were collected through in-depth individual interviews using a semistructured interview guide [17]. Participants were asked about their Internet connection, training, use of tablets, how they organized their tablet interface, experiences with the confidentiality of using tablets, and whether they had other experiences learning how to use tablets. All interviews were audio-recorded and transcribed verbatim.

### 2.5. Analyses

Data were analyzed using traditional content analysis [23, 24]. Analyses included repeated examinations of the text: first, the authors read the transcribed text from the interviews several times to obtain a sense of the content. Two researchers began coding the material to reach a common understanding of the meaning units, condensed meaning, and code designation. Then, the researchers analyzed coded material for relevance under each category. Next, the nursing material, followed by the patient material, was coded. Materials were compared for novel information, which led to a draft of categories. All researchers reviewed the saturation of categories and subcategories and discussed the results considering the research question, theory, and previous research.

## 3. Results

The analysis revealed two main categories,* network connectivity and tablet usability* and* training and educational pitfalls,* as well as four subcategories (Table 1).

### 3.1. Network Connectivity and Tablet Usability

This category had two subcategories.

#### 3.1.1. Connection Issues

In all three municipalities, OCNs and patients faced some obstacles connecting the tablets to the Internet. The OCNs experienced external IT assistance to be very helpful and they connected their tablets to the Internet easily when they followed instructions provided by the project's IT technician. OCNs also highlighted some problems with online connectivity of the patients' tablets due to the use of various networks in their homes. Connection problems came from firewalls and virus problems; patients also experienced this problem. “*It was difficult because of the firewall that we could not get through*” (patient). Some patients were given online access with help from family members or the project IT employee. One patient did not have network access installed in their home. With a simcard that was installed in the patients' tablets, network connections were possible; however, such network access could be unstable over time. There was poor network coverage in some rural areas. One patient reported, “*…on the day they came with the tablet...just then there was no net…that was strange, because it worked before they came*.”

#### 3.1.2. The Usability of VC on Tablets

When the technology worked properly, the VC on tablets positively contributed to contact between patients and nurses. Initially, the nurses were present in the patients' homes, where they handed over the tablet and provided VC training. Later, OCNs called the patients on the tablet from their office to test the web contact, voice quality, and picture quality. The usability of VC was then tested with varying amounts of conversations.

For one patient, only two VC sessions were completed because the patient avoided using the tablet. Other patients found the tablets to be easy and usable. The most frequent testing of usability occurred with several conversations weekly between the patient and the OCN during the project period.

Some patients felt that having the OCNs available was a beneficial feature: “*Just a quick conversation... I noticed when I got access to VC on a tablet that (availability) was so great.*” The technology made it possible for patients to see whether the nurses were available for a call. One patient checked on the nurses' availability every morning.

One OCN stated, “*We have somehow tried to have a systematic online sign when we are available. It lights up green*.”

For patients who preferred VC on tablets to the telephone, communication that included sound and picture was experienced as if one were talking with the OCN face-to-face. VC also helped a patient with hearing loss understand what the nurse was saying by reading nonverbal clues: “*I hear a bit poorly; it's good to be able to see the person also.*” Using VC, OCNs also received the benefit of patients' nonverbal expressions, and they could, for instance, see the patients' skin. The latter was helpful, since one patient had wounds: “*Sometimes, I had some rashes and the OCN immediately saw what my condition was.*”

One of the OCNs experienced VC with a patient who was on vacation. VC was helpful in her contact with her patient.

“Then I was on Skype with one of them when he was on vacation…he had gotten a bad message while he was on that trip. He had been on a medical checkup. The VC was nice to use then…My experience with VC was better than using a phone. The best would have been a meeting at his home, but when this was not possible, VC was a better option than just talking on the phone.”

This situation explores the usability of VC on tablet.

### 3.2. Training and Educational Pitfalls

This category comprised two subcategories.

#### 3.2.1. Importance of Previous Experiences with VC on Tablets

The amount of previous experiences with VC on tablets varied among both nurses and patients. They had varied experiences of Internet use and VC on tablets, as well as varying views on VC as a natural communication tool. Two of the four nurses were unfamiliar with use of VC on tablet computers while one had used VC to some extent, and one was familiar with VC on tablets.

Of the patients, two out of the six had used VC on tablets and one of those was familiar with the use. Three were familiar to some extent with the use of Internet and one had used a tablet computer before. The OCNs worked with older patients over 70 years of age who were less experienced with the use of the Internet than the younger ones. Several of the patients had family members who gave them support for connection and use of the Internet. One patient who only had experience with paying bills over the Internet recalled support from a grandchild: “*... just calling one of the grandchildren....”*

#### 3.2.2. Inadequate Individualized VC Training

There was no individual mapping of the need for training among either nurses or patients. The IT technician for the project who met the nurse participants at their office gave the OCNs one to two hours of individual training. He was also available for questions by both phone and network. Two nurses trained on the use of VC on tablets by communicating with each other:

“We had a quick tryout here—me and another OCN—because the other one had used VC before, and I had not used it that much. But it was easy to learn, and I quickly got into it when I began to try it out.”

The other two nurses did not have any VC training before starting VC on tablets with patients.

“...it is a little unusual; so I think it's much easier for me to take the phone and call—because I'm used to that … and it certainly has something to do with age in a way, because I think that young people are much more accustomed to communicating online.”

Regarding patient education, two nurses started the training with the patients soon after Internet access was in place, while one of the nurses took a long time before starting training. She reported that she spent a considerable amount of time teaching one patient how to use VC. Another patient with no Internet experience avoided use of the tablet computer even though she had received training. Patients who had used a tablet or computer to pay their bills found it easy to learn how to use Skype on the tablet. One of the oldest patients, who had little experience using electronic devices, stated: “*It is very easy to use such a tablet computer; it is just pressing the buttons.*” She grew very fond of the tablet.

## 4. Discussion 

Regarding the technical nature of the VC, while there were some occurrences of unstable network access, it generally functioned well. VC on tablets contributed positively to the contact between patients and nurses. Nurses and patients had very varying previous experience of Internet use and VC on tablets, but there was no mapping of individual training needs. When considering introducing VC on tablets, the research findings point to technical and human needs for quality work. Quality work should focus on measures at both technical and professional levels, as well as on patients and their needs [5, 6].

### 4.1. Network Connectivity and Usability of VC on Tablet

Technological challenges must be solved at an organizational level [5], because they can affect the communication between patient and health personnel [25]. Health personnel have emphasized the importance of well-functioning technology, which is required in order to have a usable VC encounter [26]. Even in 2018, in Norway, we found that broadband Internet, which is necessary for the use of high-quality VC, might be less available in some rural areas, similar to rural areas of Australia and the US [27, 28]. Patient safety as a structural factor in ensuring quality of health services requires secure networking [15].

After the training, VC on tablet was considered easy to use and participants were satisfied with it, which was consistent with a prior study [21]. Seen from the participants' perspective, VC was experienced satisfactorily by both patients and nurses. This was despite some problems with Internet connections. The existence of sound and picture together makes communication more comprehensive compared to just sound.

### 4.2. Training and Educational Pitfalls

The study demonstrated that there were varying types of experiences with VC on tablets among both nurses and patients. The results revealed that training and educational pitfalls revolved around participants' previous experiences with VC, as well as with the individualization of VC training. New skills that will be required are the use of tablets.

Securing user competence is a key organizational factor [5]. The study showed that it can be a challenge for some nurses and their patients to use tablets because of their lack of experience with electronic communication tools. Using computers may require mass training; therefore, nurses should participate in organized training before using VC. They should also receive guidance on how to effectively implement VC with their patients.

Some OCNs felt that they had insufficient experience in using telehealth. They needed more training than other OCNs who had such experience. Nevertheless, all OCNs saw telehealth as a facilitator, rather than a barrier, for communication [29].

Our results revealed that patients enjoyed knowing nurses' availability. Easy accessibility to healthcare personnel is of critical importance to patients living with cancer in rural areas [27, 30]. Further, the quality of the picture and sound is vital for efficient communication [31, 32]. When introducing VC as an aid in nursing, nurses should be aware of quality assurance.

### 4.3. Work Changes and Organizational Quality Assurance Work

Implementing VC on tablet as a new tool implies work changes, and accordingly new qualitative indicators that can be measured and evaluated should be developed [7].

Patient safety must always be safeguarded. The fact that patients cannot always connect to VC can be a threat to care quality. Problematically, nurses might assume that a patient is doing well simply because he/she is not reaching out to the nurse through VC; however, the silence may be caused by a poor network connection, not a lack of need. These drawbacks regarding the quality of care require further consideration.

Adopting VC on tablets for communication between patients with cancer living at home and their nurses can improve primary health service in rural municipalities. However, the implementation of quality safeguards is required before introducing VC.

### 4.4. Study Limitations

In sum, gathering data from both patients and nurses provided rich information. However, this study had a small sample size of ten participants including only two men. The study was conducted in three rural Norwegian municipalities. The results are not generalizable, and further studies should include more participants from more widely varied areas. Reliability and validity are endeavored through accurate work and collaboration between three researchers throughout the entire research process.

Risk assessments regarding the use of such a communication system were not conducted. A counterweight to this was nurses' awareness of privacy matters: they did not use the tablets in rooms where other people were present or could be listening.

## 5. Conclusion

When planning VC implementation, organizational leadership should consider network access and stability, as well as individualized training for VC on tablets. Ensuring patient safety should also be a priority. Further research should provide knowledge of technological and educational drawbacks, as well as possible implications of VC on the quality of care in nursing.

## Figures and Tables

**Table 1:** Experiences using videoconferencing during primary care.

**Category**	**Sub-category**
Network connectivity and tablet usability	Connection issues
	The usability of VC on tablet
Training and educational pitfalls	Importance of previous experience with VC on tablets
	Inadequate individualized VC training

## Data Availability

The dataset used in this study is available from the corresponding author upon reasonable request and in consideration of ethical concerns.
